# Transcriptomic Coupling of PKP2 With Inflammatory and Immune Pathways Endogenous to Adult Cardiac Myocytes

**DOI:** 10.3389/fphys.2020.623190

**Published:** 2021-01-18

**Authors:** Marta Pérez-Hernández, Grecia M. Marrón-Liñares, Florencia Schlamp, Adriana Heguy, Chantal J. M. van Opbergen, Valeria Mezzano, Mingliang Zhang, Feng-Xia Liang, Marina Cerrone, Mario Delmar

**Affiliations:** ^1^The Leon H. Charney Division of Cardiology, New York University Grossman School of Medicine, New York, NY, United States; ^2^Genome Technology Center, Department of Pathology, New York University Grossman School of Medicine, New York, NY, United States; ^3^Microscopy Laboratory, Division of Advanced Research Technologies, New York University Grossman School of Medicine, New York, NY, United States

**Keywords:** arrhythmogenic cardiomyopathy, plakophilin-2, inflammation/immune response, transcriptome, GTEx

## Abstract

Plakophilin-2 (PKP2) is classically defined as a component of the desmosome. Besides its role in cell–cell adhesion, PKP2 can modulate transcription through intracellular signals initiated at the site of cell–cell contact. Mutations in *PKP2* associate with arrhythmogenic right ventricular cardiomyopathy (ARVC). Recent data demonstrate that inflammation plays a key role in disease progression; other results show an abundance of anti-heart antibodies in patients with confirmed diagnosis of ARVC. Here, we test the hypothesis that, in adult cardiac myocytes, *PKP2* transcript abundance is endogenously linked to the abundance of transcripts participating in the inflammatory/immune response. Cardiac-specific, tamoxifen (TAM)-activated PKP2-knockout mice (PKP2cKO) were crossed with a RiboTag line to allow characterization of the ribosome-resident transcriptome of cardiomyocytes after *PKP2* knockdown. Data were combined with informatics analysis of human cardiac transcriptome using GTEx. Separately, the presence of non-myocyte cells at the time of analysis was assessed by imaging methods. We identified a large number of transcripts upregulated consequent to PKP2 deficiency in myocytes, inversely correlated with PKP2 abundance in human transcriptomes, and part of functional pathways associated with inflammatory/immune responses. Our data support the concept that PKP2 is transcriptionally linked, in cardiac myocytes, to genes coding for host-response molecules even in the absence of exogenous triggers. Targeted anti-inflammatory therapy may be effective in ARVC.

## Introduction

Plakophilin-2 (PKP2) is classically defined as a component of the desmosome, a structure of the cardiac intercalated disc (ID). Desmosomes are intercellular adhesion complexes and serve as anchoring point for intermediate filaments at the cell end. In addition to cell–cell adhesion, PKP2 and its ID partners translate information initiated at the site of cell–cell contact into intracellular signals that modulate transcription (ID–transcription coupling) (Cerrone et al., 2017, 2019; Montnach et al., 2018; Kim et al., 2019). Here, we examine the transcriptional relation between PKP2 and the cardiac inflammatory and immune responses.

*PKP2* mutations associate with most cases of gene-positive familial arrhythmogenic right ventricular cardiomyopathy (ARVC) (Basso et al., 2009; Groeneweg et al., 2015; Philips and Cheng, 2016), a disease characterized by fibrofatty infiltration of right ventricular (RV) predominance, ventricular arrhythmias, and high propensity for sudden death in the young. Most often, the disease begins with a subclinical, concealed stage, followed by overt stages of RV predominance (though left ventricle is often involved) and then bi-ventricular dilated cardiomyopathy progressing to end-stage failure and heart transplant or death (Basso et al., 2009; Groeneweg et al., 2015; Philips and Cheng, 2016; Austin et al., 2019). While the clinical phenotype of ARVC is extensively characterized, molecular mechanisms underlying the cardiomyopathy remain poorly understood.

It has long been suspected that inflammatory and immune responses are part of the ARVC landscape. ARVC is known to “flare” in some patients, in a manner reminiscent of viral myocarditis (Pieroni et al., 2009). Recently, Chelko et al. (2019) demonstrated the key role that inflammation may play in disease progression. A separate study showed the abundance of anti-heart antibodies in patients with confirmed diagnosis of ARVC (Caforio et al., 2020). The question remains as to whether PKP2 and the inflammatory/immune response are transcriptionally linked in adult cardiac myocytes, in such a way that PKP2 deficiency endogenously (without a pathogen as trigger) activates a transcriptional program that can lead to a “sterile myocarditis” phenotype. We addressed this question using a combination of experimental and analytical methods. We identified a large number of transcripts upregulated consequent to PKP2 deficiency in murine myocytes, inversely correlated with PKP2 abundance in the human transcriptome, and a part of functional pathways associated with inflammatory/immune responses, including those related to viral infections. Our data support the concept that PKP2 is transcriptionally linked, in cardiac myocytes, to genes coding for host-response molecules even in the absence of an exogenous trigger.

## Materials and Methods

All experimental procedures conformed to the Guide for Care and Use of Laboratory Animals published by the US National Institutes of Health and approved by the NYU-IACUC committee under protocol number 160726-03.

### Generation of Cardiac-Specific RiboTag/PKP2 Knockout Mice

A C57BL/6 RiboTag^*flox*^ mice line was purchased from The Jackson Laboratory (011029 B6N.129-*Rpl22^TM 1^.^1*Psam*^*/J). RiboTag^*flox/flox*^ mice possess loxP sites flanking exon 4 of ribosomal protein L22 (RPL22). Crossing of RiboTag mice with cre-expressing mice yields offspring where exon 4 of RPL22 in the cre-expressing tissue is substituted by a triple HA-tagged version of the same exon (RPL22-3HA). RiboTag^*flox/flox*^ mice were crossed with PKP2^*flox/flox*^//αMHC-Cre-ER(T2) [αMHC-Cre-ER(T2)] codes for the α myosin heavy chain promoter and for the ligand binding domain of the human estrogen receptor (Cerrone et al., 2017), to obtain (RiboTag^*flox/flox*^)/(PKP2^*flox/flox*^)/Cre+. For the differential transcriptome analysis, (RiboTag^*flox/flox*^)/(PKP2^*wt/wt*^)/Cre + animals were used as controls (i.e., precipitation of mRNA from the myocytes of mice expressing PKP2). The genotypes of RiboTag^*flox/flox*^ mice were identified by PCR reaction with the loxP primers (forward: GGG AGG CTT GCT GGA TAT G; reverse: TTT CCA GAC ACA GGC TAA GTA CAC). For αMHC-Cre-ER(T2) genotyping, the forward primer TTATGGTACCACATAGACCTCTGACA and reverse TGCTGTTGGATGGTCTTCACAG were used.

Mice were born at expected genotype ratios and developed normally. Mice of both genders, 3 months of age, were intraperitoneally injected four consecutive days with tamoxifen (TAM) (3 mg dissolved in sterile peanut oil with 10% ethanol, giving an approximate TAM dose of 0.1 mg/g body weight) to activate the Cre-recombinase and induce both the cardiomyocyte-specific Cre-mediated deletion of PKP2 and the expression of the 3HA-tagged gene. All procedures conformed to the Guide for Care and Use of Laboratory Animals published by the US National Institutes of Health and were approved by the NYU IACUC committee under protocol number 160726-03.

### Immunoprecipitation of RNA From RiboTag/PKP2cKO Mice and Controls

RNA was extracted from six RiboTag control and five PKP2cKO/RiboTag mice hearts 21 days post-injection (dpi) of TAM. To prepare RNA immunoprecipitation beads, 100 μl protein G Dynabeads (Thermo Fisher) were washed once in citrate-phosphate buffer (PB) pH 5.0 (24 mM citric acid and 52 mM dibasic sodium phosphate); buffer was separated and removed from the beads by using Dynabeads magnet. Subsequently, the beads were incubated on a rotational platform for 4 h at 4°C with 10 μl mouse monoclonal anti-HA antibody (BioLegend) in 200 μl citric buffer at pH 5.0. After incubation, the beads were washed once with citrate buffer, followed by wash in Tris buffer pH 7.5 (50 mM Tris pH 7.5, 100 mM KCl, 12 mM MgCl_2_, 1% Nonidet P-40, 1 mM DTT, 200 U RNAase inhibitor, 1 mg/ml heparin, 100 μg/ml cycloheximide, and protease inhibitor). After the final wash, the homogenate (∼400 μl in Tris pH 7.5) of RiboTag/PKP2cKO or control heart was applied to the beads and rotated overnight at 4°C.

The HA antibody-bound RNA was extracted using RNeasy Plus Micro Kit (QIAGEN 74034). Briefly, after overnight incubation, the beads were washed once gently in 50 mM Tris pH 7.5 and then vortexed shortly in 400 μl RLT buffer with βME. The supernatant was transferred to a gDNA Eliminator Spin Column and then centrifuged for 30 s at 8000 × *g*. The flow-through was mixed with 400 μl 70% ethanol and purified using RNeasy MinElute column. The resulting RNA samples were analyzed by NanoDrop and confirmed for RNA integrity number (RIN) > 8 by BioAnalyzer prior to RNA-seq analysis.

### RNA-Seq Analysis

RNA library preps were made and sequenced as paired-end mode at the Genome Technology Center at NYU using Illumina HighSeq2500. Approximately 31.97 million reads per sample were generated. Quality control (QC) for the RNA-seq reads was assessed using FastQC and MultiQC software (Ewels et al., 2016). Low-quality base noise in the beginning of the reads was trimmed using FASTX-Toolkit fastx_trimmer^1^. Next, the two reads per sample (R1 and R2) were mapped to the mouse reference genome (build GRCm38/mm10) using Spliced Transcripts Alignment to a Reference (STAR version 2.6.1) (Dobin et al., 2013). Sequence duplicates were removed using picard-tools MarkDuplicates^2^ (version 2.18.0). Genomic features were then assigned using featureCounts (Liao et al., 2014). The read counts of each transcript were normalized using DESeq2 package in R (version 3.6.0) (Love et al., 2014). In order to validate the intra-group consistency, a principal component analysis was performed. Gene expression differences were evaluated using the Wald test after data normalization. The *p*-values were corrected via the Benjamini and Hochberg method. We defined as “differentially expressed” those genes with a log2 fold change of at least ± 0.5 between a pair of samples and a *p*-adjusted under 0.05 (FDR < 5%). For differentially expressed genes, we performed functional and biological process enrichment analysis using DAVID^3^ (Huang et al., 2009) and Enrichr^4^ (Kuleshov et al., 2016) software. We looked for enrichment for genetic association with the Kyoto Encyclopedia of Genes and Genomes (KEGG) pathway database and Gene Ontology (GO) annotations. Moreover, the oPOSSUM software^5^ (Kwon et al., 2012) was used for the detection of over-represented conserved transcription factor (TF) binding sites.

### PKP2 Expression Analysis in Human Left Ventricle

We used RNA-seq data obtained from the left ventricle of 386 deceased human donors. The raw data were retrieved from the Genotype-Tissue Expression database v8 (GTEx)^6^. Genes were filtered by removing the ones with no transcripts per million (TPM) in <50% of samples and genes TPM < 1 in >5% of samples. To ensure read count normalization and homoscedasticity, we performed the variance stabilizing transformation, as implemented in the R package DESeq2 (version 1.26.0). Co-variants such as gender, age, and RIN were used in the linear regression analysis. The cut off for PKP2 was set to the following: regression coefficient β < −0.5 and -log(*p*-value) > 10. For the analysis of overlapping transcripts (present both in PKP2-GTEx and the RiboTag RNA-seq), thresholds for analysis inclusion were β < −0.5 and -log(*p*-value) > 10. All GTEx RNA-Seq analysis was performed in R studio version 3.6.3. Genes of interest were analyzed for enrichment for genetic association using the KEGG database, with the R-package clusterProfiler (version 3.14.3) (Yu et al., 2012).

### Weighted-Gene Network Analysis

To generate the biological gene network, we used the R-based weighted correlation network analysis (WGCNA) package (version 1.69) (Langfelder and Horvath, 2008). First, a gene co-expression network was formed and represented by an adjacency matrix. Then, highly interconnected genes (modules) were grouped together by hierarchical clustering. To measure the dissimilarity between modules, WGCNA uses the topological overlap measure (TOM). To obtain moderately large and distinct modules, we set the minimum module size to 20 genes and the minimum height for merging modules at 0.25. The modules were randomly color-labeled. Un-clustered genes (genes not assigned to any module) were labeled in gray and not used for further analysis.

### Immunohistochemistry

Control and PKP2 cKO 21 dpi mice were anesthetized by inhalation of 100% CO_2_. After deep anesthesia was confirmed by lack of response to otherwise painful stimuli, mice were euthanized by cervical dislocation. The heart was surgically excised, perfused with 2% paraformaldehyde, kept in the same fixative solution at 4°C overnight, and paraffin embedded. Five-micron sections of paraffin-embedded tissue were stained with Akoya Biosciences Opal Multiplex Automation Kit reagents unless stated otherwise. Automated staining was performed on Leica Bond RX autostainer. The protocol was performed according to manufacturers’ instructions with the antibodies listed in Table 1. Briefly, all slides underwent sequential epitope retrieval with Leica Biosystems epitope retrieval 2 solution (EDTA-based, pH 9, Cat. AR9640); primary and secondary antibody incubation; and tyramide signal amplification with Opal fluorophores Op620, Op570, and Op520. Primary and secondary antibodies were removed during epitope retrieval steps while fluorophores remain covalently attached to the tissue. Semi-automated image acquisition was performed on a Vectra 3.0 multispectral imaging system. After whole-slide scanning at 10×, the tissue was manually outlined to select fields for multispectral imaging at 20×. inForm version 2.4.2 software from Akoya Biosciences was used for spectral unmixing and image analysis.

**TABLE 1:** Immunohistochemistry antibodies (AB).

Antibody	Company	Host	Clone	Catalog	Dilution
Vimentin	EMD Milipore	Chicken	Polyclonal	AB5733	1:200
Anti-CD45	Abcam	Rat	Monoclonal	ab25386	1:250
Ly-6G/Ly-6C	BioLegend	Rat	Monoclonal	108402	1:200
Anti-Chicken Alexa 488	Thermo Fisher	Goat	Polyclonal	A-11039	1:100
Rat-on-Rodent HRP-Polymer	Biocare Medical	Not applicable		BRR4016	1:150
Rabbit-on-Rodent HRP-Polymer	Biocare Medical	Not applicable		RMR 622	1:400

### Serial Block Face Scanning Electron Microscopy

#### Sample Preparation

Control and PKP2cKO 14 dpi mice were anesthetized by inhalation of 100% CO_2_, perfused with 4% paraformaldehyde, and then euthanized by excision of the heart. The perfused heart was cut into 1 mm^3^ and placed in a fixative solution containing 2% paraformaldehyde and 2.5% glutaraldehyde in PB (pH 7.2). The fixed mouse hearts were processed by a modified OTTO procedure and embedded in Durcupan. In brief, the heart tissue was washed with 0.1 M PB, post fixed in 2% OsO_4_/1.5% potassium ferrocyanide for 1.5 h at room temperature, and then stained with freshly made 1% tannic acid (Electron Microscopy Sciences, EMS, Hatfield, PA, United States) in PB for two consecutive steps with 2 h each step at 4°C to allow for additional staining. The tissue was then washed in ddH_2_O, placed in 2% aqueous OsO_4_ for 40 min at room temperature, and en bloc stained in 1% aqueous uranyl acetate at 4°C overnight. The tissues were then washed with ddH_2_O, dehydrated in a series of ethanol solutions (30, 50, 70, 85, 95, and 100%; 10 min each, on ice), and replaced with ice-cold dry acetone for 10 min, followed by 10 min in acetone at room temperature. The sample was gradually equilibrated with Durcupan ACM Araldite embedding resin (Electron Microscopy Sciences, EMS, Hatfield, PA, United States) and embedded in freshly made 100% Durcupan.

#### Electron Microscopy

The sample block was trimmed and thin sections were cut on slot grids to identify the area of interest. The sample block was then mounted on an aluminum specimen pin (Gatan, Pleasanton, CA, United States) using silver conductive epoxy (Ted Pella Inc.) to electrically ground tissue block. The specimen was trimmed again with pyramid shape and coated with a thin layer of gold/palladium (Denton Vacuum DESK V sputter coater, Denton Vacuum, LLC, Moorestown, NJ, United States). Serial block face imaging was performed using Gatan OnPoint BSE detector in a Zeiss GEMINI 300 VP FE-SEM equipped with a Gatan 3View automatic microtome unit. The system was set to cut sections with 90-nm thickness, imaged with nitrogen gas injection setting at 40% (2.9E-03mBar) with Focus Charge Compensation to reduce the charge, and images were recorded after each round of section from the block face using the SEM beam at 1.2 keV with a dwell time of 2.0 μs/pixel and a working distance of 7.4 mm. Each frame is 38 × 50 μm with a pixel size of 4 nm. Data acquisition occurred in an automated way using the Auto Slice and View G3 software to collect a 1 × 3 montage scan with a 15% (7.5 μm) overlap. A stack of 150 slices was aligned, stitched, and assembled using the Grid/Collection stitching plugin for ImageJ (Preibisch et al., 2009). A volume of 38 × 105 × 15 μm^3^ dimensions was obtained from the tissue block.

#### Image Segmentation

The epicardium was segmented in Amira (Visage Imaging, San Diego, CA, United States) to generate 3D models.

## Results

### PKP2cKO/RiboTag Mice to Study the PKP2-Dependent Cardiomyocyte Transcriptome

**FIGURE 1:**
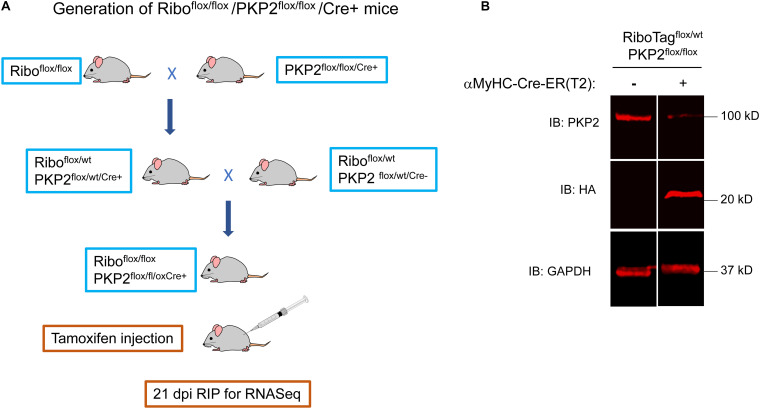
Generation of Ribo^*flox/flox*/^PKP2^*flox/flox*/*Cre+*^ mice. **(A)** RiboTag^*flox/flox*/^-PKP2^*flox/flox*^ mice were obtained crossing RiboTag mice (JAX B6N.129-Rpl22/J 011029) with PKP2cKO/αMyHC-Cre-ER(T2). Cre + and Cre- mice were tamoxifen-injected and used 21 days post injection (dpi). **(B)** Mice hearts were analyzed by western blot. The 23-kDa 3HA epitope-tagged ribosomal protein RPL22 was detected by an HA antibody. A heterozygous RiboTag^*flox/wt*^ was used. For experiments, we used homozygous RiboTag^*flox/flox*^ to increase 3HA-RPL22 expression and facilitate breeding scheme.

Characterization of PKP2-dependent cardiac transcriptomes has been limited to whole-heart samples, which include cells of different types, not only myocytes. To determine the transcriptome specific to cardiomyocytes, we crossed PKP2cKO mice (Cerrone et al., 2017) with a previously validated and commercially available murine line called “RiboTag” (details in section “Materials and Methods”; see Figure 1A). Active Cre-recombinase leads to expression of an RPL22 that is modified in exon 4 to code for a 3-repeat HA tag (3HA). RPL22-3HA is expressed and incorporated into the ribosome particle, allowing its precipitation and subsequent detection of associated transcripts by RNA-seq. Given that Cre-ERT2 expression in our mice was controlled by the αMHC promoter, injection of TAM led to both, loss of PKP2 expression and expression of RPL22-3HA. We refer to this line as PKP2cKO/RiboTag. The western blot in Figure 1B shows loss of PKP2 protein and expression of RPL22-3HA genes 21 days after TAM injection in PKP2cKO/RiboTag mice. Immunoprecipitation of RPL22-3HA and subsequent RNA-seq provided a snapshot of mRNA in the translational machinery of cardiac myocytes at the time of isolation. To determine the PKP2cKO vs control transcriptome, “control” mice were PKP2^*wt/wt*^, RiboTag^*flox/flox*^, and Cre-positive. Controls and PKP2cKO/RiboTag mice were injected with TAM on the same day.

### PKP2-Dependent Transcriptome of Adult Murine Cardiac Myocytes

**FIGURE 2:**
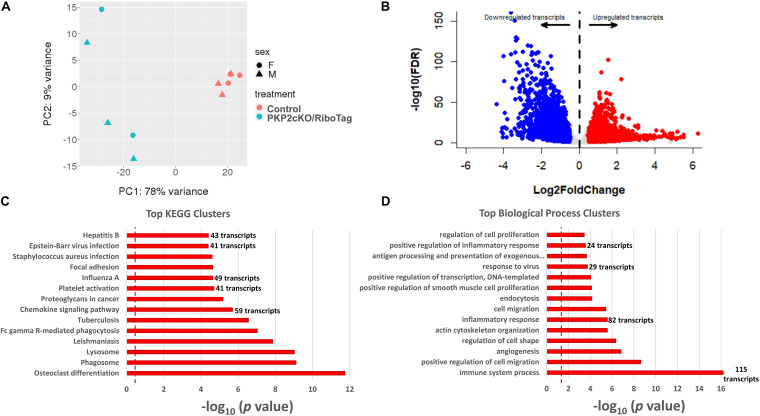
Upregulated transcripts in hearts of PKP2cKO/RiboTag mice. **(A)** Principal component analysis (PCA) based on RNA-seq of myocyte control or PKP2cKO/RiboTag mice. The 78% of variance can be explained by treatment (PKP2 knockdown) whereas sex explained only 9% of variance, allowing data to be combined. **(B)** Volcano plot representing differential transcriptome of PKP2cKO/RiboTag myocytes vs control. “Upregulated” (red) or “downregulated” (blue) refers to abundance in PKP2 knocked-down myocytes (control as reference). Inclusion criteria are as follows: Log2 fold change ± 0.5 and FDR < 0.05. **(C)** Significantly enriched Kyoto Encyclopedia of Genes and Genomes (KEGG) pathways from upregulated genes. **(D)** Significantly enriched biological process clusters from upregulated genes in PKP2cKO.

We obtained a complete transcriptome (RNA-seq) from six controls and five PKP2cKO/RiboTag hearts. Principal component analysis revealed segregation of two populations based on genotype, and no segregation based on sex (Figure 2A). Thus, to increase statistical power, samples from both sexes were pulled together. Figure 2B shows a volcano plot of the differential transcriptome. Samples were enriched for myocyte-specific transcripts (Supplementary Figure I; list of transcripts in Supplementary Table 1). Using thresholds for log2 fold change ± 0.5 and FDR < 0.05, we obtained 3186 transcripts differentially downregulated and 3157 upregulated by loss of PKP2 (Supplementary Table 1). KEGG and GO biological processes of upregulated transcripts (Figures 2C,D, respectively) showed over-representation of transcripts involved in platelet activation and chemokine signaling, viral response, inflammatory, and immune pathways (Figures 2C,D).

### Non-myocyte Cell Infiltration in PKP2cKO Ventricular Tissue

**FIGURE 3:**
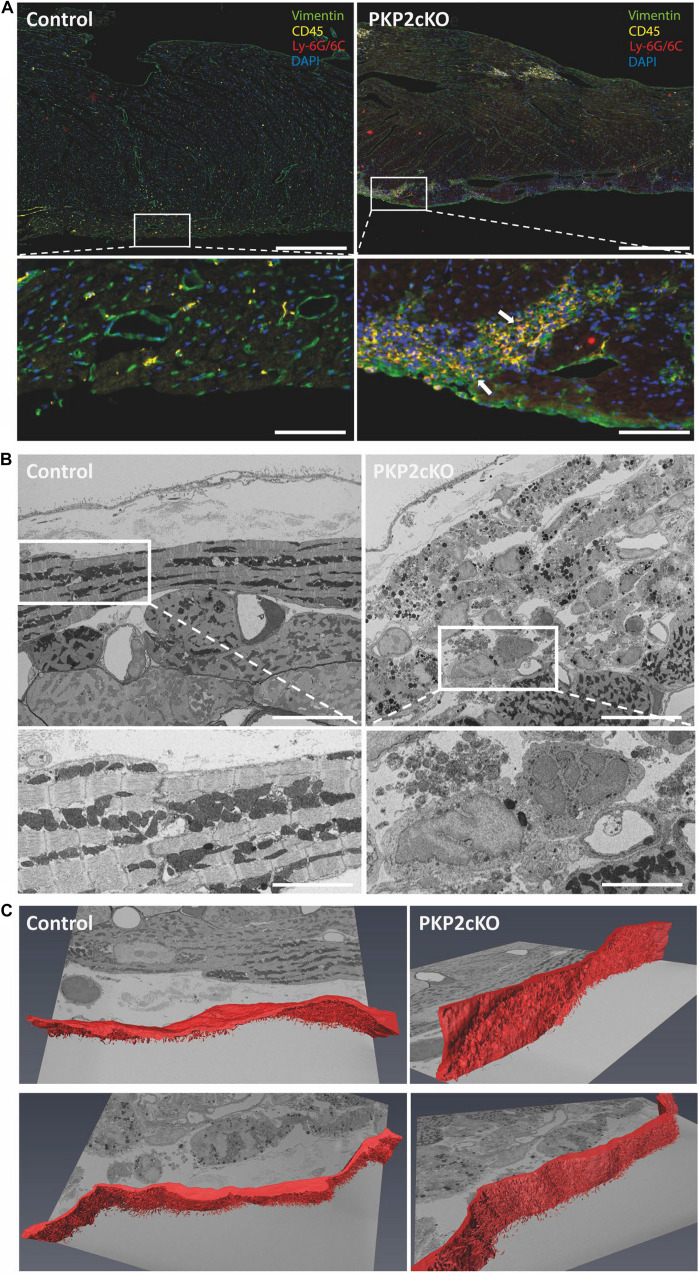
Non-myocyte cell infiltration in ventricular tissue of wildtype and PKP2cKO mice. **(A)** Opal Multiplex immunohistochemistry staining for vimentin (green), CD45 (yellow), Ly-6G/Ly-6C (red), and DAPI (blue) in control and PKP2cKO ventricular tissue 21 days post-tamoxifen injection (dpi). Scale bar = 200 μm. White insets depict zoomed-in view; arrows highlight infiltration of CD45 and Ly-6G/6C-positive cells in PKP2cKO subepicardium. Scale bar = 50 μm. **(B)** Serial block face scanning electron microscopy (SBF-SEM) images of control (left) and PKP2cKO (right) subepicardial ventricular tissue, 14 dpi. Upper left panel (control), top to bottom: pericardial space; epicardial cell layer; basal membrane; first myocytes aligned parallel to epicardial surface. In right panel (PKP2cKO), see abundance of subepicardial non-myocyte cells and injured myocytes. A first layer of “healthy” myocytes is seen in bottom right corner. Scale bar = 10 μm. White frames indicate areas enlarged in bottom. Scale bar = 5 μm. **(C)** Single frames of complete z-stack of SBF-SEM images, with segmentation analysis of epicardial layer (red). Complete stack is in Supplementary Movies.

The transcriptional changes observed in myocytes at this stage (21 days post-TAM; see Cerrone et al., 2017) coincided with the presence of infiltrates positive for T-cell marker CD45 and for neutrophil marker Ly-6G/Ly-6C (Figure 3A). Using serial block face scanning electron microscopy (SBF-SEM), we observed, at an earlier stage (14 days post-TAM) (Kim et al., 2019), non-myocyte cells in the subepicardial region of the myocardium (Figures 3B,C). Of note, at this stage in disease progression, histological changes were not yet apparent (see Kim et al., 2019).

### PKP2 Inverse Correlation Analysis in Human Left Ventricle

**FIGURE 4:**
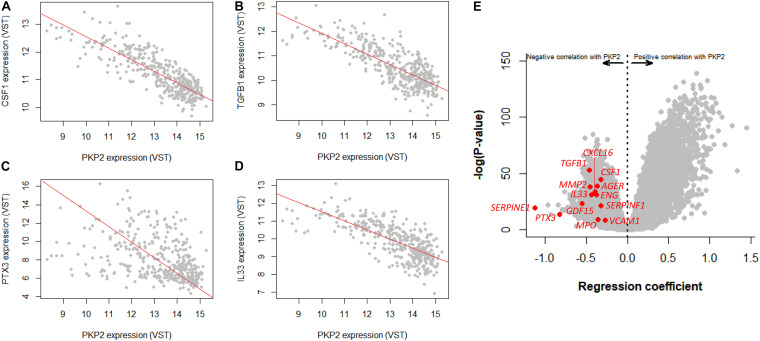
GTEx-based correlation analysis of human left ventricle transcriptome in relation to PKP2 expression. **(A–D)** Linear regression analysis of a transcript (CSF1, TGFB1, PTX3, and IL33, respectively) vs PKP2 expression. Notice negative slope for the four transcripts, all related with inflammation and immune response. Each gray point represents normalized level of PKP2 vs that of the given transcript for each individual in the 386 dataset. **(E)** Repeating this process for PKP2 against the entire transcriptome generates a volcano plot. Specific transcripts related to inflammation/immune system are highlighted in red. VST, variance stabilizing transformation.

**FIGURE 5:**
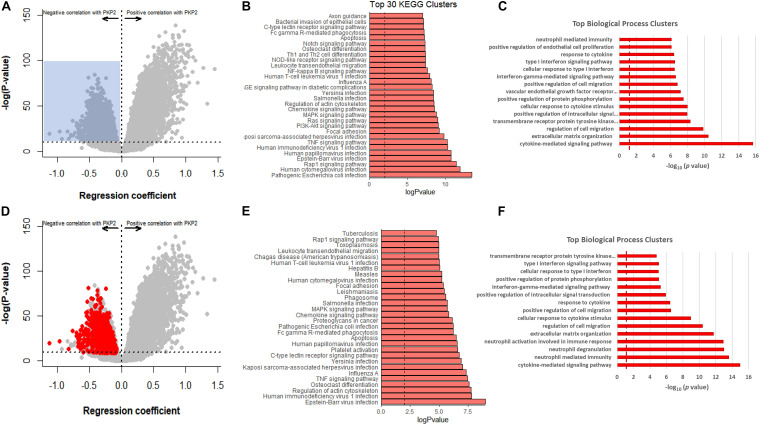
GTEx-based PKP2 transcript correlation analysis, in comparison to RNA-seq PKP2cKO/RiboTag. **(A)** Correlation analysis of PKP2 normalized transcript abundance against other transcripts in the GTEx left ventricle database. Dots in gray are the same as in Figure 4E. Blue shadow indicates area selected for pathway analysis (regression coefficient < −0.5 and *p*-adjusted > 1E-10. **(B,C)** Top 30 Kyoto Encyclopedia of Genes and Genomes (KEGG) pathways and biological process analysis, respectively, of transcripts inversely correlated with PKP2. **(D)** Same plot as **(A)** but red circles indicate 912 transcripts significantly upregulated in RiboTag RNA-seq dataset. **(E,F)** Top 30 KEGG pathways and biological process analysis of red transcripts.

Infiltration of non-myocyte cells may in turn affect the differential transcriptome obtained from the myocytes. To further examine the intrinsic association between PKP2 and other cardiac transcripts, we took advantage of the GTEx v8 database, where transcript abundance correlations can be obtained between one specific transcript (PKP2 in our case) and all others in the transcriptome of the left ventricle of 386 human decedents. Plots in Figures 4A–D explain the data process. Each point in the plot corresponds to the relative abundance of PKP2 (abscissa) and a given transcript (e.g., CSF1, TGFB1, PTX3, and IL33 in Figures 4A–D) in a given individual. A linear regression was calculated for the total data set (386 data points; one for each case). The resulting slope and statistical significance of the linear regression were plotted separately (abscissae and ordinates, respectively), each transcript contributing a single point (Figure 4E). Repeating the process for all 17,434 transcripts in the transcriptome produces a complete volcano plot (Figure 4E). Negative numbers in the abscissae indicate an inverse correlation (a negative slope of the linear regression; see Figures 4A–D), i.e., the lesser the abundance of PKP2 transcripts, the more the abundance of the correlated transcript. We extracted a list of transcripts inversely correlated with PKP2 with a regression coefficient (β) < −0.5 and with a *p*-adjusted value higher than 1E-10 (complete list in Supplementary Table 2). Functional pathways based on these transcripts revealed, as in the case of PKP2cKO/RiboTag, a predominance of pathways related to viral infection, platelet activation, inflammation, and immune response networks (Figures 5A–C).

### Inverse Correlation Analysis of PKP2-Dependent Genes

The GTEx data only present correlations, rather than a relation ascribable to PKP2 abundance. On the other hand, the PKP2cKO/RiboTag data provide us with a list of transcripts that change consequent to PKP2 loss. We therefore extracted transcripts significantly and inversely correlated with PKP2 in the PKP2cKO/RiboTag data and localized those in the GTEx data plot (Figure 5D). A total of 912 significant transcripts were identified through this process (dataset in Supplementary Table 3). As in the case of the transcriptome data of PKP2cKO/RiboTag and of GTEx correlative analysis, functional pathways from overlapping datasets were consistent with inflammation and immune responses, in particular with pathways associated with viral infections (Figures 5E,F).

**FIGURE 6:**
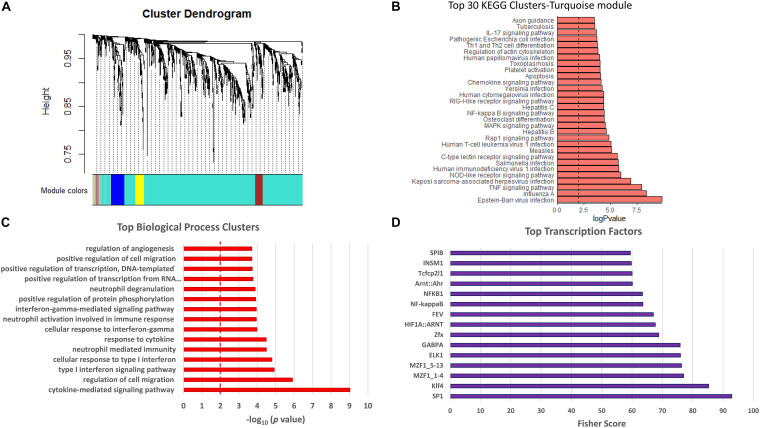
Weighted gene co-expression network analysis (WGCNA). Data included were the same as those in red in Figure 5D, i.e., significantly upregulated in RNA-seq dataset of PKP2cKO/RiboTag and negatively correlated with PKP2 expression in GTEx. **(A)** Cluster dendrogram of genes from WGCNA analysis, with dissimilarity based on topological overlap. Four modules detected and assigned to colors turquoise, blue, brown, and yellow, with number of genes ranging from 39 to 740. **(B)** Top 30 Kyoto Encyclopedia of Genes and Genomes (KEGG) pathways from the most abundant module (turquoise). **(C)** Top biological process associated with transcripts in the most abundant module (turquoise). **(D)** Transcription factor analysis for genes in turquoise module.

The dataset in Figure 5D and Supplementary Table 3 was used to conduct weighted gene correlation network analysis (WGCNA) (Langfelder and Horvath, 2008). A total of four modules were identified, and each was assigned to a color: turquoise, brown, blue, and yellow (Figure 6A, Supplementary Figure II, and Supplementary Table 4). The primary and most abundant module (turquoise) included 740 transcripts. Again, inflammation and immune response transcripts, chemokine signaling pathways and platelet activation, and pathways associated with viral infections were most prominent (Figures 6B,C).

The list of transcripts inversely correlated to PKP2 in the GTEx dataset and upregulated after PKP2 knockdown in mice hearts was examined for their relation to specific TFs using the oPOSSUM program (Kwon et al., 2012). The identity of TFs most closely linked to genes in Supplementary Table 3 is presented in Figure 6D. Notice the presence of GABPA, NFkB, NFkB1, SP1, and SPIB, all related to inflammatory and immune responses (Lee et al., 2005; Gallant and Gilkeson, 2006; Perdomo-Sabogal et al., 2016; Liu et al., 2017).

## Discussion

Several investigators have identified inflammatory infiltrates in hearts of patients with ARVC (Campuzano et al., 2012; Chelko et al., 2019). This observation could be explained as consequent to loss of myocyte integrity and activation of a reparative mechanism. From that perspective, myocytes would be seen only as initiators, once engaged in apoptosis or cell death, of the expression of inflammatory/immune response transcripts in non-myocyte cells. The recent work of Chelko et al. (2019) strongly suggested that myocytes are not only triggers but also participants in the production of cytokines that are part of the molecular profile of ARVC. Here, we build on that observation to show that there is a relation between abundance of PKP2 transcripts and expression, by cardiomyocytes, of transcripts coding for proteins involved in inflammatory and immune responses. The GTEx data further show this relation in hearts not afflicted with ARVC. In other words, this is an intrinsic relation, endogenous to heart cells, rather than secondary to a primary cardiac pathology.

Intercellular communication between myocytes and non-myocyte cells may be a component of the transcriptional response observed in PKP2cKO hearts. While this possibility is likely, our data from the GTEx analysis indicate that the relation between PKP2 transcript abundance and the abundance of transcripts involved in the inflammatory/immune response can be documented independently from the existence of a cardiac disease phenotype. As such, our data do not exclude the possibility of a cross-talk between intercellular and intracellular mechanisms; our data seek to better document the latter, following on the breakthrough studies of Chelko et al. (2019), as well as others. How the intercellular and the intracellular pathways interact with each other remains a matter of further study.

Our findings do not show that the association in transcript abundance is directly established by PKP2 (i.e., PKP2 acting as TF). Though this could be the case, there is no evidence for direct regulation of transcription by PKP2. Previous investigators have shown PKP2 fragments in the nucleus of cells in culture; yet, their presence in adult myocytes has neither been demonstrated nor is there evidence of PKP2 binding to DNA in adult cardiomyocytes. On the other hand, PKP2 abundance can affect the localization and function of ID proteins that affect transcription, particularly beta-catenin (Chen et al., 2014), plakoglobin (Li et al., 2011), and PKC-alpha (Bass-Zubek et al., 2008). Coupling between PKP2 and transcription is likely mediated by multiple factors rather than (or in addition to) direct involvement of PKP2 as TF.

Previous studies showed that loss of PKP2 in murine hearts can lead to increased abundance of transcripts related to paracrine regulation (Cerrone et al., 2018). A novel aspect of our study is the use of RiboTag to select transcripts from adult cardiomyocytes. This method has been validated extensively (Sanz et al., 2019); here, we adapt it to study PKP2-mediated dysregulation. Importantly, RiboTag allows for a snapshot of ribosome-associated transcriptomes at the time of the experiment. It is therefore possible that the method is skewed toward genes associated with a high transcription rate and against others for proteins of long half-lives and low transcription rates. From that perspective, we acknowledge that differences in transcript abundance do not necessarily mean equal differences in protein abundance. That being said, in separate studies, we have found good correlation between changes in transcript and changes in protein content (Cerrone et al., 2017; Montnach et al., 2018).

We emphasize that our experimental model (PKP2cKO) neither is intended to “recapitulate” ARVC nor is intended to be a surrogate for studies of human hearts affected with disease. No experimental model, be it murine or cell-based, properly recapitulates ARVC. Differences between cells in murine hearts or in a dish and a human heart are fundamental. Experimental models do not reproduce the ecosystem of a human heart with an ARVC mutation. What we do in our experiments is not to mimic (or “recapitulate”) ARVC. We identify the cardiac endophenotype of a protein that, when mutated in a human heart, causes ARVC. Extrapolation from our data to the specific case of ARVC needs to be done with caution. That said, our model has shown consistency with human informatics data (Cerrone et al., 2017; Montnach et al., 2018) and has been useful in proposing therapeutic approaches to patients with ARVC (e.g., the pilot clinical trial on use of flecainide in ARVC patients; NCT03685149).

Cardiomyocytes are highly specialized and, as such, primarily studied for their electro-mechanical function. Yet, they retain their ability to produce, release, and sense chemical factors for interaction with other cells, myocytes or non-myocytes. We identified transcriptomic pathways coincident with response to viral or bacterial infections. Notably, a number of these pathways link to viruses or bacteria known to produce myocarditis, such as Epstein–Barr, HIV-1, influenza A, herpes virus, or Salmonella (Caforio et al., 2013). The degree of convergence strengthens the notion that alterations in PKP2 can yield some (not all) of the clinical manifestations of viral myocarditis and that this response can be independent of the actual presence or exposure to a pathogen. It also argues that if indeed there is a viral infection, activation of similar networks may be part of the molecular mechanisms responsible for myocarditis. Overall, we show that PKP2 transcriptionally activates pathways that can mimic phenotypes reminiscent of myocarditis without necessarily being activated by a pathogen, in other words, a sterile myocarditis.

We acknowledge that the GTEx dataset is gathered from samples that contain RNA from myocyte and non-myocyte cells. As such, in the GTEx analysis, the correlation between one gene and another may reflect associations that pertain, or not, to the specific case of myocytes. It is precisely because of this that we combine the GTEX analysis with the analysis of the RiboTag data, where samples are indeed collected from myocytes. The fact that the datasets converge gives credence to the notion that the GTEx results reflect, at least in part, gene networks that would be present in the myocytes. We also recognize that the GTEx hearts are not sick and, as such, important differences would be expected, as the enhancement of neutrophil activation pathways observed in the RiboTag data, which is not apparent in the GTEx.

Analysis of transcripts upregulated in PKP2cKO/RiboTag mice and negatively correlated with PKP2 identified the “C-type lectin receptor signaling” as a prominent pathway. C-type receptors induce production of inflammatory cytokines and chemokines, consequently triggering innate and adaptive immunity. Transcripts associated with this pathway are mainly part of the Dectin−1 signaling that induces differentiation of T-helper cells Th1 and Th17 (Leibundgut-Landmann et al., 2007). These cells are a source of cytokines that participate in the “cytokine storm.” Th17 produces IL-17, which plays a crucial role in viral infections and, synergistically with IL6, facilitates virus persistence by limiting apoptosis of virally infected cells (Hou et al., 2014). Th17 and IL17 associate with myocarditis, including its autoimmune form (Bockstahler et al., 2020), and with inflammatory cardiomyopathy (Myers et al., 2016). Overall, these correlations link to the significant presence in our dataset of NFkβ, REL, and EGR1, TFs related with Th17 differentiation and immune response (Ruan et al., 2011).

There is a tight connection between fibrinolysis, platelet activation, and immune system (Schuliga, 2015). Of note, three transcripts shared in the PKP2 and significantly upregulated in PKP2cKO/RiboTag mice are *Plau*, *Plaur*, and *Serpine1*. *Serpine1* codes for plasminogen activator inhibitor 1 (PAI-1) (Schuliga, 2015). In ARVC, although thromboembolic complications are not common (Wlodarska et al., 2006), myocyte necrosis perhaps caused by micro-thrombus is a possible cause of myocardial injury (Pilichou et al., 2009). Moreover, PAI-1 is a well-known target of TGFβ (whose transcript is also increased in our PKP2cKO/RiboTag model) and fundamental in TGFβ-induced fibrosis (Schuliga, 2015). A relation between PAI-1 and Wnt/beta-catenin signaling, a pathway likely relevant to ARVC pathophysiology (Lorenzon et al., 2017), has been shown (He et al., 2010).

The presence of NFkB as an associated TF linked to PKP2 further highlights the importance of recent studies of Chelko et al. pointing at NFkB as potential target for ARVC therapy. In this regard, drugs that target the immune/inflammatory response may help treat the “sterile myocarditis” occurring in ARVC patients.

## Data Availability Statement

The datasets presented in this study can be found in online repositories. The names of the repository/repositories and accession number(s) can be found below: BioProject ID PRJNA684590.

## Ethics Statement

The animal study was reviewed and approved by the NYU-IACUC committee under protocol number 160726-03.

## Author Contributions

MP-H and GM-L were responsible for the day-to-day operation of the project and participated in its design, carried out the informatics analysis, and participated in the writing of the manuscript. FS processed the RNA-seq data for analysis. AH was instrumental in generating the RNA-seq raw dataset. CO analyzed the electron microscopy images under the guidance of F-XL. VM performed the immunofluorescence experiments. MZ generated the PKP2cKO/RiboTag mice and was responsible for collecting the RNA samples for analysis. MC and MD conceived and funded the study, supervised the research, and participated in the writing of the manuscript with input from all authors. All authors contributed to the article and approved the submitted version.

## Conflict of Interest

The authors declare that the research was conducted in the absence of any commercial or financial relationships that could be construed as a potential conflict of interest.

## Footnotes

1 http://hannonlab.cshl.edu/fastx_toolkit/

2 http://broadinstitute.github.io/picard

3 https://david.ncifcrf.gov/summary.jsp

4 https://amp.pharm.mssm.edu/Enrichr/

5 http://opossum.cisreg.ca/oPOSSUM3/

6 https://www.gtexportal.org/home/
