# Supplementary material for: Transcriptomic Coupling of PKP2 With Inflammatory and Immune Pathways Endogenous to Adult Cardiac Myocytes
Source: Front Physiol. 2021 Jan 18;11:623190. doi: 10.3389/fphys.2020.623190 (PMC7849609; doi:10.3389/fphys.2020.623190)

Supplementary Material

1. Supplementary Figures legends

Supplementary Figure I. Enrichment of cardiomyocyte-specific genes in the PKP2cKO/RiboTag model. A. Boxplot showing the raw counts for each positive control gene (cardiomyocyte-specific: *Tnnt2*, *Myh7*) and each negative control gene (epicardial-specific: *Aldh1a2*, *Tbx18*, *Tcf21*, *Wt1*). **B**. Boxplot showing the normalized counts for each positive control gene and each negative control gene.

Supplementary Figure II. **Weighted gene co-expression network analysis (WGCNA) of transcripts in red from figure 5D** (transcripts significantly up-regulated in the RNA-Seq dataset of PKP2cKO/RiboTag mouse hearts, and negatively correlated with PKP2 expression in GTEx)**: blue, brown and yellow modules**. **A**. Top image: Hierarchical clustering dendrogram of module eigengenes (labeled by their colors). Heatmap plot of the adjacencies in the eigengene network is represented below. Each row and column in the heatmap corresponds to one module eigengene (labeled by color). In the heatmap, blue color represents low adjacency (negative correlation), while red represents high adjacency (positive correlation). **B.** KEGG pathway analysis of the blue module. **C.** KEGG pathway analysis of the yellow module. **D.** Biological process analysis of the blue module. **E.** Biological process analysis of the yellow module **F.** Biological process analysis of the brown module. No KEGG pathways were found for the brown module.

2. Supplementary Excel Table legends

Supplementary Table 1. Complete transcriptome (RNA-seq) from PKP2cKO/RiboTag vs Control murine hearts.

Supplementary Table 2. List of human transcripts (GTEx) that inversely correlated with PKP2 with a regression coefficient (β) < -0.5 and with a *p*-adjusted value higher than 1E-10.

Supplementary Table 3. List of 912 transcripts significantly and inversely correlated with PKP2 in the PKP2cKO/RiboTag data and localized in the PKP2-GTEx data plot.

Supplementary Table 4. List of transcripts in each of the four detected WGCNA module.

3. Supplementary Movie legends

Supplementary video 1 and 2. Serial block face-scanning electron microscopy (SBF-SEM)-resolved three-dimensional ventricular subepicardial ultrastructure from control and plakophilin-2 conditional knockout (PKP2cKO) mice respectively.

Supplementary Figure I

A

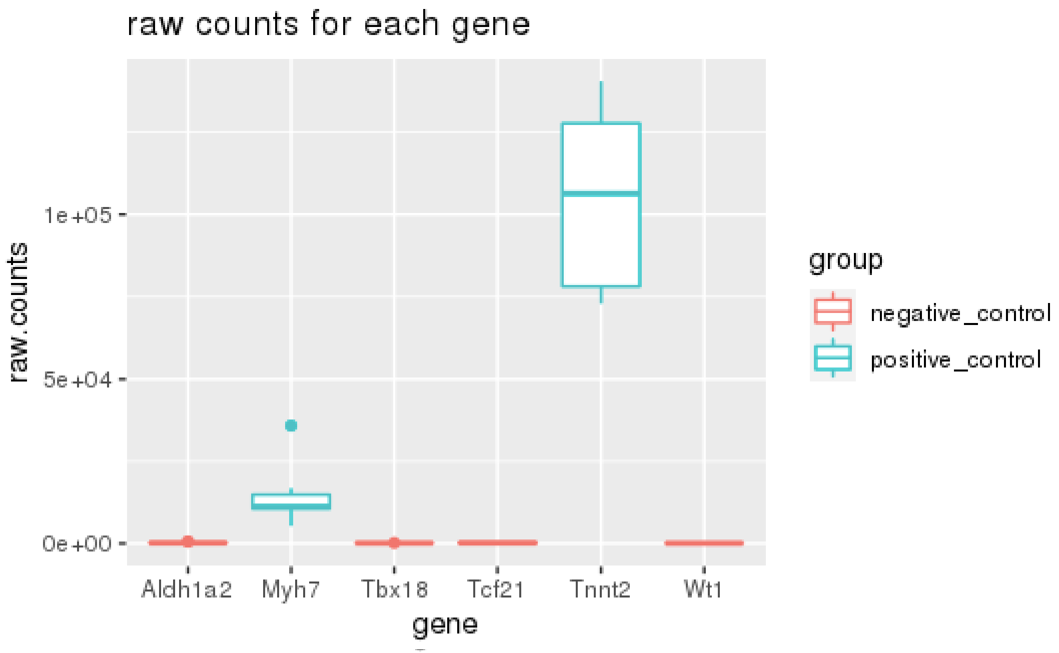

B

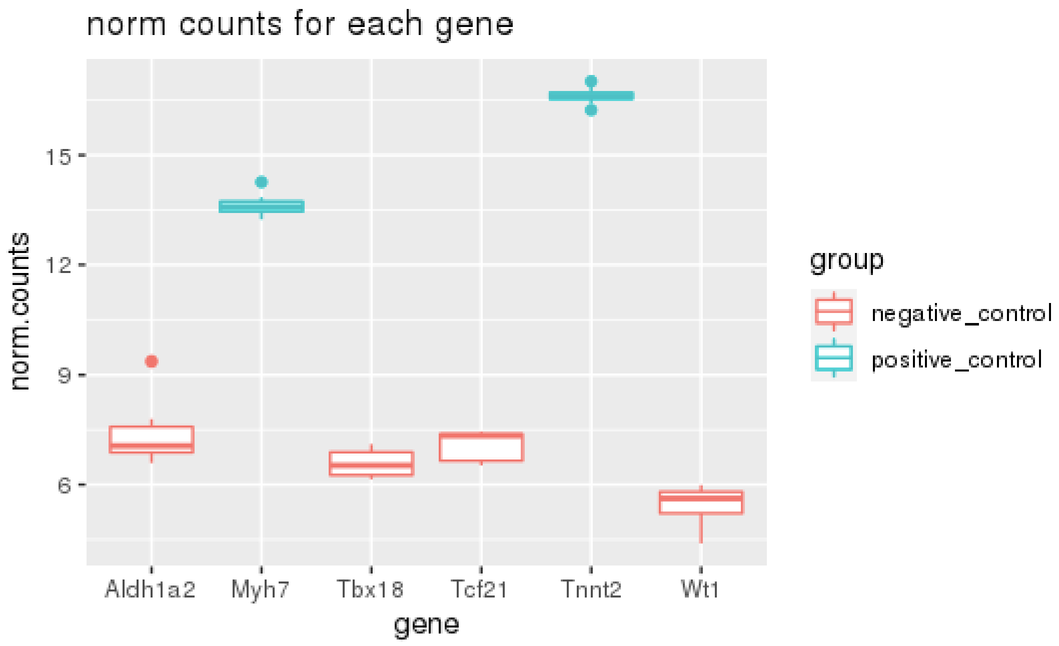

Supplementary Figure II

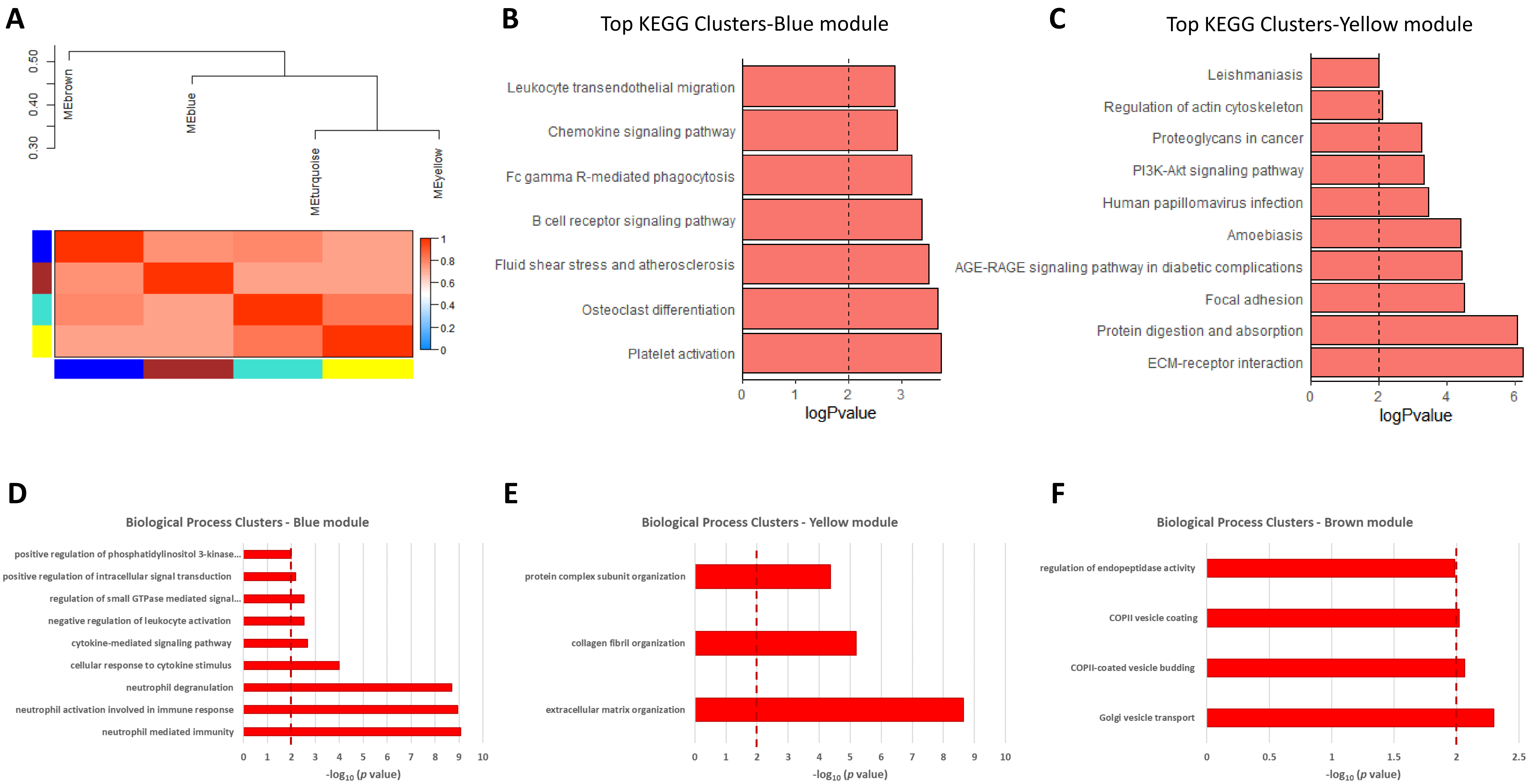

Supplement: Supplementary file 1 [file Data_Sheet_1.pdf]
